# Designing Aerosol Therapies Based on the Integrated Evaluation of In Vitro, In Vivo, and In Silico Data

**DOI:** 10.3390/pharmaceutics15061695

**Published:** 2023-06-09

**Authors:** Margaret Bourlon, Yu Feng, Lucila Garcia-Contreras

**Affiliations:** 1 Department of Pharmaceutical Sciences, University of Oklahoma Health Science Center, Oklahoma City, OK 73104, USA; 2 School of Chemical Engineering, Oklahoma State University, Stillwater, OK 74078, USA

**Keywords:** pulmonary drug delivery, aerosol formulation, aerosol performance, in vitro/in vivo correlation, in silico modeling, computational fluid dynamics

## Abstract

Despite the advantages of the pulmonary route of administration and inhalable dosage forms, other routes of administration and dosage forms are often considered first to treat lung diseases. This occurs, in part, due to the perceived limitations of inhaled therapies resulting from the improper design and interpretation of their in vitro and in vivo evaluation. The present study outlines the elements that should be considered in the design, performance, and interpretation of the results of the preclinical evaluation of novel inhaled therapies. These elements are illustrated using an optimized model poly(lactic-co-glycolic) acid (PLGA) microparticle (MP) formulation to optimize the site of MPs deposition. The different expressions of MP size were determined, and their aerosol performance in devices used for animal (Microsprayer^®^ and Insufflator^®^) and human studies (nebulizer and DPIs) was assessed using inertial impaction. Radiolabeled MPs were delivered to the lungs of rats by spray instillation to determine their site of deposition using single-photon emission computed tomography (SPECT) imaging. Recommendations to optimize the in vitro determinations are given, as well as suggestions to evaluate and interpret in vivo data in the context of the anatomy and physiology of the animal model and the corresponding in vitro data. Recommendations for the proper selection of in vitro parameters to inform in silico modeling are also given, as well as their integration with in vivo data.

## 1. Introduction

The advantages of pulmonary drug delivery to treat asthma, chronic obstructive pulmonary disorder [1,2], and the respiratory symptoms of cystic fibrosis [3,4,5] have been detailed many times over [6,7,8], but compared to other pharmaceutical dosage forms, inhaled formulations are rarely used. Some of the reasons for the smaller number of products for inhalation include the additional cost of the inhaler device (nebulizer and compressor, metered dose inhaler, or dry powder inhaler), the need for specialized formulations, and the equipment to produce them in industrial quantities. Another factor that discourages using an inhalable dosage form for novel therapeutic compounds is the misconceptions about their efficacy and effectiveness. This occurs particularly at small or start-up companies or in the academic setting, where novel compounds are often developed or discovered by basic science researchers. In these settings, investigators in the initial stages of projects involving novel compounds are often experts on the disease they are aiming to treat, but they may have limited experience in the formulation of aerosol therapies and their in vitro and in vivo characterization. Many times, the use of a less-than-optimal formulation, the incomplete in vitro characterization, and the lack of understanding of these results to design proper in vivo studies are the reason for pursuing other routes of administration or abandoning further development of promising compounds for lung diseases.

The design and development of any aerosol drug-delivery system are highly complex because the formulation must be matched with a suitable device for optimal inhaled therapy [9]. An ideal inhaler-formulation combination should deliver precise and consistent doses to a targeted region in the lungs and maintain the stability of the delivered drugs [10,11]. To overcome the limitations that traditional drug-carrier formulations impose on the performance of DPI devices, sophisticated particle engineering technologies have been developed to produce stable powders that are less dependent on the device and have improved aerosol performance and bioavailability [12,13,14].

The in vitro evaluation of the aerosol drug-delivery system in preclinical studies can be conducted in several ways, and each method yields different information (e.g., geometric, volume, and aerodynamic (MMAD) diameters) about the particles or droplets that form the aerosol being studied. One of the main limitations of integrating in vitro data with in vivo studies is the incorrect interpretation of the in vitro data to design in vivo studies, particularly if only one or two of these methods are used. For example, if only the geometric diameter is determined for an aerosol, it would not be possible to determine if there is particle aggregation or hygroscopic growth. In contrast, if only the volume diameter is determined, it would not be possible to observe the shrinkage of liquid droplets or the breaking of particles due to shear forces. A study developing microparticles for the treatment of pulmonary arterial hypertension characterized the size of five tacrolimus microparticle formulations by their geometric diameter and aerosol performance and found that three of the formulations had an MMAD > 5 µm [15]. However, based only on their geometric diameter instead of MMAD, they assumed that all five formulations would deposit in the deep lung and further tested all formulations in cell culture instead of just the three that had the suitable MMAD. Another misconception about pulmonary drug delivery is that all devices are created equal. The drug delivery device must be carefully selected for each formulation by determining the aerosol performance of the inhalable formulation in several devices: for example, low-resistance and high-resistance DPIs, or jet nebulizers versus vibrating mesh nebulizers. For instance, in a study that developed several inhalable formulations containing rapamycin and berberine to treat lung cancer, the size of microparticles was characterized by their geometric diameter and aerosol performance using a DPI [16]. The authors then used these data to select a formulation for studies in mice but used a dry powder insufflator to administer the powders without considering the difference in airway sizes from humans to mice; thus, it is likely that the deposition pattern was different in the in vivo study. More importantly, if the therapeutic activity of a compound depends on the integrity of its molecular structure, as is the case for proteins and peptides, the activity of the compound should be evaluated before and after its aerosolization. For example, a study evaluating the safety, tolerability, and immunogenicity of an aerosolized adenovirus-based COVID-19 vaccine employed a vibrating mesh nebulizer to immunize the patients and concluded that the aerosolized vaccine had comparable performance to the I.M. injection [17]. In this study, the researchers determined the volume median diameter of the nebulized vaccine, but they did not determine its aerosol performance to estimate the dose delivered to each patient. Moreover, the viability of the vaccine after nebulization was not evaluated to ensure that the aerosolization did not affect its protective efficacy. Without these two determinations, it is not possible to discern if the dose or the integrity of the vaccine negatively influenced the result of the study. In contrast, Jeyanathan et al. performed a detailed and complete characterization of the nebulized droplets of a TB vaccine but found that only 17.4% of the administered dose was viable after treatment [18]. Nevertheless, it would have been valuable to evaluate other nebulizers or different delivery devices to preserve the viability of the vaccine.

Additional challenges in preclinical studies with inhaled therapies are the selection of the animal model to conduct early efficacy studies and the selection of a device to deliver the aerosol to these animals in a manner relevant to humans. The advantages and disadvantages of the different animal models to evaluate inhaled therapies, as well as the different methods of aerosol administration, have been described in detail previously [19,20]. However, the manner or the context in which the results of these animal studies should be interpreted so that they are relevant to therapies in humans are still not clear. For instance, what is the correct interpretation of deposition studies performed in rodents to design therapies in humans, given that the diameter of their airways is different? In addition, this interpretation is even more difficult when the efficiency of aerosol therapies depends on the stage of the disease. For example, it is known that the diameter of the lumen of the airways of cystic fibrosis (CF) patients is reduced by the stage of the disease, but for a while, deposition data in healthy subjects was used to design inhaled therapies in CF patients. Fortunately, there have been elegant in silico models to describe the effect of narrowing airways on the site of aerosol deposition that has guided the modification of the methods of aerosol generation [21,22]. This is not the case for other pulmonary diseases such as TB. Even though it is known that the integrity of the airway structure in infected patients is greatly affected by the stage of the disease, there are no guidelines yet that would direct the improvement of the efficacy of inhaled therapies to treat TB. These two cases (CF and TB) illustrate the importance of adding in silico methods such as computational fluid dynamics (CFD) and discrete element method (DEM) to explicitly predict and visualize the sites of aerosol deposition [23,24] and how the disease stage would affect them.

The purpose of this study is to provide a basic overview of items to be considered in the design, performance, and interpretation of in vitro and in vivo studies to assess the suitability of novel compounds to treat pulmonary diseases. Practical examples of the key in vitro determinations that should be performed on inhalable formulations and their integration with in vivo and in silico determinations are provided using model microparticles.

## 2. Materials and Methods

### 2.1. Materials

Poly-D,L-lactide-co-glycolide (PLGA; 75:25, MW 76,000–115,000 kd, intrinsic viscosity 0.90 dL/g), Nile red dye, and Tween 80 were purchased from Sigma–Aldrich (St. Louis, MO, USA). All solvents were of HPLC grade and were purchased from Sigma–Aldrich. Medical grade saline was purchased from Braun (B. Braun Medical Inc., Bethlehem, PA, USA). Compressed Nitrogen (N_2_) gas was purchased from Airgas (Oklahoma City, OK, USA).

### 2.2. Optimization of the Manufacturing Parameters of Microparticles (MPs)

To prepare blank microparticles (MPs), PLGA (200–350 mg) was dissolved in methylene chloride (MeCl) and spray dried with a Buchi-290 mini spray dryer (Buchi Labortechnik AG, Flawil, Switzerland) in an open-loop configuration. Spray drying parameters were optimized to produce particles in respirable sizes (1–5 µm) using Design of Experiments software (DoE, Design Expert, Stat-Ease, Inc., Minneapolis, MN, USA). The following parameters were entered into a 2^3^ full factorial design: feed concentration (0.25–0.35%), nitrogen gas flow rate (600–742 L/h), and aspiration rate (50–75%). The feed flow rate (7.5 mL/min), atomization pressure (3.0 bar), and inlet temperature (65 °C) were kept constant. A subsequent DoE was used to refine the 3D size of MPs by entering the following manufacturing parameters into a 2^2^ full factorial design: aspiration rate (50–75%) and feed flow rate (3.0–7.5 mL/min). Atomization pressure (3.0 bar), inlet temperature (65 °C), feed concentration (0.25%), and nitrogen (N_2_) gas flow rate (742 L/h) were kept constant in this design (DoE2).

After optimization of the manufacturing parameters to produce the desired size and distribution of blank MPs, the same parameters optimized in DoE1 and DoE2 for blank MPs were used to prepare MPs containing Nile red dye to be used in inertial impaction determinations. The dye was dissolved into the PLGA/MeCl solution (1% *w*/*v*) before spray drying.

#### 2.2.1. Entrapment Efficiency of Nile Red Dye

The entrapment efficiency of Nile red in the MPs was determined by first dissolving a known mass of PLGA-Nile red MPs in MeCl, and then adding methanol (MeOH) to precipitate the PLGA leaving the dye in solution (1-part MeCl to 9-parts MeOH). Subsequently, the sample was centrifuged for 30 min at 15,000 RPM (Eppendorf centrifuge 5424). The absorbance of the supernatant was quantified using a UV spectrometer (Lambda 25, PerkinElmer Inc., Waltham, MA, USA) with a wavelength set at 552 nm. A calibration curve was prepared for Nile red using a concentration range of 0.5–25 µg/mL.

#### 2.2.2. Confirmation of Solvent Removal in the Final PLGA Microparticles

The presence of residual MeCl in different samples was determined using a Nicolet X Summit Attenuated total reflection Fourier-Transform Infrared Spectroscope with a diamond crystal (ThermoFisher, Waltham, MA, USA) [25]. Two drops of MeCl were placed on the crystal and covered with a cap to minimize evaporation during analysis. Dry powders of unprocessed PLGA, 1:1 PLGA:MeCl, and optimized PLGA MPs were also placed on the crystal as a thin layer (1 µm) for analysis. FTIR spectra were analyzed using OMNIC^®^ Specta software (ThermoFisher, Waltham, MA, USA).

### 2.3. Physicochemical Characterization of MPs

#### Morphology, Particle Size, and Distribution

The morphology and geometric diameter (Dg) of the MPs were determined from images of the MPs obtained by scanning electron microscopy (SEM, Zeiss Neon 40 EsB). For this purpose, a small mass (<5 mg) of MPs was obtained from the jar of the spray dryer and gently deposited on double-coated carbon conductive tape (Ted Pella Inc., Redding, CA, USA) mounted on aluminum stubs suitable for the SEM. Each sample was sputter coated (Anatech Ltd., Battle Creek, MI, USA, Hummer VIO) with gold-palladium (5–6 nm) before being imaged. At least five images were taken from each powder batch, and at least 50 particles were measured from each of these images. Individual particles from each image were measured using ImageJ software (version 1.53d, National Institutes of Health). At least 300 particles were measured for each batch of the DoEs. The particle size versus cumulative frequency data was plotted on a log probability paper to determine the Dg and its corresponding standard deviation (GSD_Dg_).

The volume diameter (Dv) of MPs in the bulk powder and its corresponding standard deviation (GSD_Dv_) were determined using a HELOS laser diffraction system (Sympatec Inc., Lawrenceville, NJ, USA) and the RODOS accessory. The RODOS dispersion pressure was varied (0.5–2.0 bar) to determine its influence on the resulting Dv and GSD_Dv_. In addition, the Dv of MPs in an aqueous suspension (saline with 0–2% Tween 80) was determined using the HELOS cuvette accessory.

The influence of the device employed to generate dry or wet aerosols on the emitted Dv was evaluated using two devices for direct administration (bypassing the nasopharyngeal region of the respiratory tract) to animals (Insufflator^®^ and MicroSprayer^®^) and two devices for passive inhalation (a nebulizer and two dry powder inhalers). To measure the size of dry and wet aerosols, a small animal Insufflator^®^ (Penn-Century model DP-4) or a MicroSprayer^®^ (Penn-Century Model IA-1B Aerosolizer) was placed and secured onto a custom-made stand designed to position the device at a distance on which the mid-region of the generated aerosol plume would be measured by the laser of the HELOS laser diffraction system. The customizable GRADIS disperser was also employed to aid in the size determination of aerosols generated by the Insufflator^®^. The same method was used to measure the Dv of droplets generated from MPs in suspension using a nebulizer (Hudson RCI© Micro Mist©). All HELOS measurements were obtained with a time base of 5 milliseconds, and detection was triggered when the optical concentration in the measuring zone exceeded 0.5%.

### 2.4. Aerosol Performance

To predict the site of deposition of PLGA-Nile red MPs aerosolized from bulk powders or MPs in suspension, using direct administration or passive inhalation methods, their aerosol performance was evaluated by inertial impaction. Dry aerosols were generated using the Insufflator^®^ (Penn-Century model DP-4), the HandiHaler DPI (SPIRIVA), and a Plastiape RS01 Monodose DPI. The wet aerosols were generated using the MicroSprayer^®^ (Penn-Century Model IA-1B Aerosolizer) and a nebulizer (Hudson RCI© Micro Mist© with Vios Pro compressor).

The aerosol performance of the MPs dispersed from each device was evaluated using a next-generation impactor (NGI, Westech) comprised of an induction port, seven stages with decreasing pore diameters, and a micro-orifice collector (MOC). Each device was attached to the induction port using a custom adaptor. A vacuum was pulled through the system at 10–80 L/min for 60 s to disperse the powder along the stages of the impactor. The induction port and collection cups for each stage were rinsed with acetonitrile, and the PLGA-Nile red content was analyzed by UV Spectrometry using the same method employed to determine entrapment efficiency.

The aerosol performance was evaluated in terms of mass median aerodynamic diameter (MMAD), geometric standard deviation (GSD_MMAD_), emitted dose (ED), and fine particle fraction (FPF). The MMAD was calculated as described in USP <601>, and the GSD_MMAD_ was calculated in the same manner as GSD_Dg_ and GSD_Dv_. The ED was calculated as:

$$Emitted\;dose\left(\%\right)=\left(\frac{PLGA\;mass\;recovered\;from\;NGI}{PLGA\;mass\;loaded\;into\;device}\right)*100$$

The FPF was calculated as the percentage of particle mass recovered below a specified cutoff diameter from the total mass of particles recovered from the NGI. For humans, the FPF was calculated using a cutoff diameter of 4.46 µm and included particles recovered from stages 3–7 and the MOC. For small rodents (rats, guinea pigs, etc.), the FPF was calculated using a cutoff diameter of 2.28 µm and included particles recovered from stages 4–7 and the MOC.

### 2.5. Technetium-99m- Labeling of PLGA MPs and IMAGING

MPs were radiolabeled with Tc-99m following a reported procedure with some slight modifications [26,27]. A 1 µg/µL saline solution of SnCl_2_ was prepared and purged with Nitrogen gas. To the 500 µL saline solution of 5 mg of dry powdered PLGA MPs in an Eppendorf tube was added 250 µL of SnCl_2_ solution. The mixture was sonicated and vortexed for 10 min, followed by the addition of 200 µL of 100% ethanol and 250 µL of 20 mCi Na^99m^TcO_4_ solution. The reaction mixture was incubated at 45 °C for 1 h with shaking. Quality control was performed on a Silica Gel TLC plate using acetone/saline (50/50 *v*/*v*) as eluent, and analysis was done on a radio-TLC detector indicating 100% labeled MPs at the origin.

### 2.6. In Vivo Lung Deposition Maps Using SPECT/CT

Image studies were performed by the OU College of Pharmacy’s Research Imaging Facility as a fee-per-service. Male Sprague Dawley rats (251–275 g, 8–10 weeks old, *n* = 5) were dosed with the 99mTc radiolabeled blank PLGA particles as follows. First, rats were anesthetized with isoflurane (4% vaporizer output), and then the trachea was visualized with the help of a small animal laryngoscope (PennCentury, Model LS-2-R) and the tube of the MicroSprayer^®^ was introduced into the trachea via the larynx. The 2 mCi of radiolabeled PLGA MPs in suspension (100 µL) were aerosolized into the rat’s lungs using a Hamilton syringe attached to the MicroSprayer^®^. After administration, a 30 min helical SPECT using a BioScan NanoSpect (Mediso, Budapest, Hungary) and a 2 min CT acquisition using a GammaMedica XO XPET (Northridge, CA, USA) were obtained. The 3D radioactivity distribution maps were generated to determine the site of MP deposition in the airways. All the animal experiments performed in this study were approved by the University of Oklahoma Health Sciences Center Institutional Animal Care and Use Committee (IACUC) under Protocol number 19-074-RX.

### 2.7. Data Analysis

The values of the size and distribution responses were analyzed with the DoE software. First, the absolute values of the effect were displayed in a half-normal probability plot, and the magnitude of each effect was confirmed in a Pareto chart. After the effects with the largest magnitude were selected, the chosen variables were analyzed with ANOVA based on an F-test (*p* < 0.05 was considered significant). The equation quantifying the outcome of each variable was built using the effects considered significant.

Average particle diameters (i.e., Dg, Dv, and MMAD) were assumed to be the diameter of particles in the 50th percentile of the distribution. The widths of the geometric deviations were calculated using one standard deviation above and below the median particle diameters according to the formula GSD = √(d_84%_/d_16%_), where d_84%_ and d_16%_ represent the diameters at the cumulative percentile of 84% and 16% of the particle size distribution after it has been “normalized”. All other statistical analyses were conducted using a one-way ANOVA and with Tukey’s multiple comparisons tests (GraphPad PRISM) unless otherwise stated. Statistical significance was set at *p* < 0.05. All values are reported as mean ± standard deviation.

## 3. Results

### 3.1. Physiochemical Characterization

The parameters of manufacture that were optimized to obtain PLGA MPs in the respirable range using DoE1, as well as their corresponding diameters and GSDs, are listed in Table 1. The Dg of the resulting particles ranged from 2.7 µm to 3.7 µm, and the GSD_Dg_ ranged from 1.48 to 1.94, whereas Dv ranged from 5.39 µm to 5.68 µm with GSD_Dv_ ranging from 1.15 to 1.2, indicating that in the bulk powder, the MPs behaved as small uniform aggregates. 

**Figure 1:**
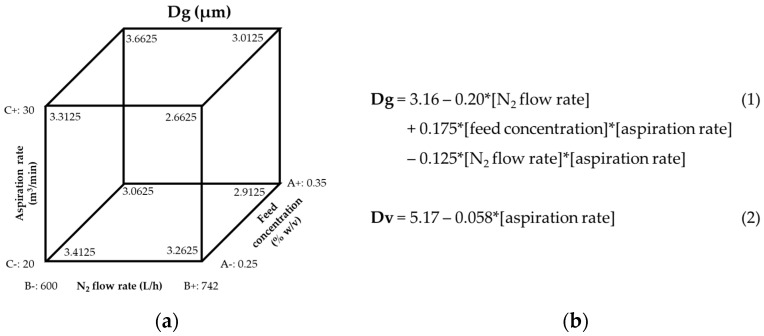
Models generated by the analysis of DoE1: (**a**) Cube plot showing the influence of spray drying parameters on the Dg of PLGA MPs, and (**b**) Equations that numerically describe the influence of manufacturing parameters on Dg (1) and Dv (2).

The cube plot in Figure 1a suggests that the feed concentration was the single parameter that influenced the Dg of the resulting MPs, but Equation (1) in Figure 1b indicates that the flow rate and the interaction between the feed flow rate and aspiration rate are the parameters that will determine the formation of smaller particles (Dg). On the other hand, Equation (2) in Figure 1b indicates that the aspiration rate is the primary spray-drying parameter that influences the sizes of MP aggregates. Based on the equations and the factor coefficients, the smallest particle sizes were obtained by increasing the N_2_ flow and aspiration rates and decreasing the feed concentration.

Based on these results, DoE2 was designed to evaluate if a decrease in feed flow rate and an increase in aspiration rate would further decrease the size of MP aggregates (Dv). Table 2 shows that, indeed, an increase in aspiration rate and a decrease in feed flow rate resulted in a modest decrease in Dv (from 5.39 µm to 5.07 µm), but that was more likely due to a comparable decrease in Dg (from 2.70 µm to 2.30 µm) rather than a reduction in MP aggregation.

Additional batches were prepared using the optimized conditions (0.25% *w*/*v* feed concentration, 7.5 mL/min feed flow rate, 742 L/h N_2_ flow rate, and 35 m^3^/min aspiration rate) to verify the reproducibility of these conditions to obtain MPs in the same sizes. These conditions were also employed to prepare MPs containing Nile red. Nile red MPs had a Dg of 2.30 µm with a GDS_Dg_ of 1.65 and a Dv of 5.12 µm with a GSD_Dv_ of 1.14, demonstrating that the incorporation of dye in the formulation did not significantly affect the sizes of the resulting MPs or the reproducibility of the spray-drying conditions. The entrapment of Nile red dye in the PLGA MPs was 111.9 ± 2.2%.

**Figure 2:**
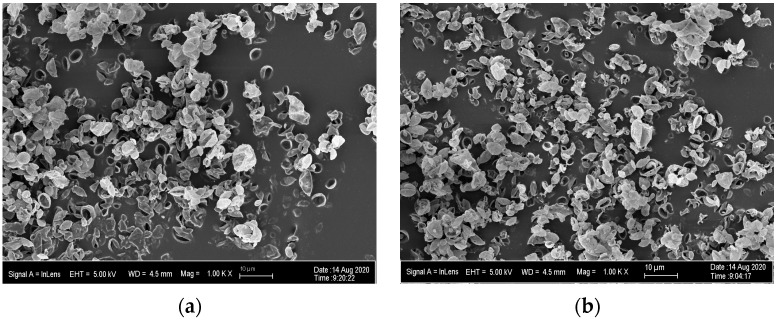
SEM images of (**a**) blank PLGA MPs and (**b**) PLGA MPs with Nile red dye manufactured with the spray drying conditions optimized and refined in the DoEs (0.25% *w*/*v* feed concentration, 7.5 mL/min feed flow rate, 742 L/h N_2_ flow rate, and 35 m^3^/min aspiration rate).

PLGA MPs resembled collapsed hollow spheres and had approximately one cavity-like depression per particle (Figure 2a). The incorporation of Nile red dye affects particle morphology (Figure 2b).

**Figure 3:**
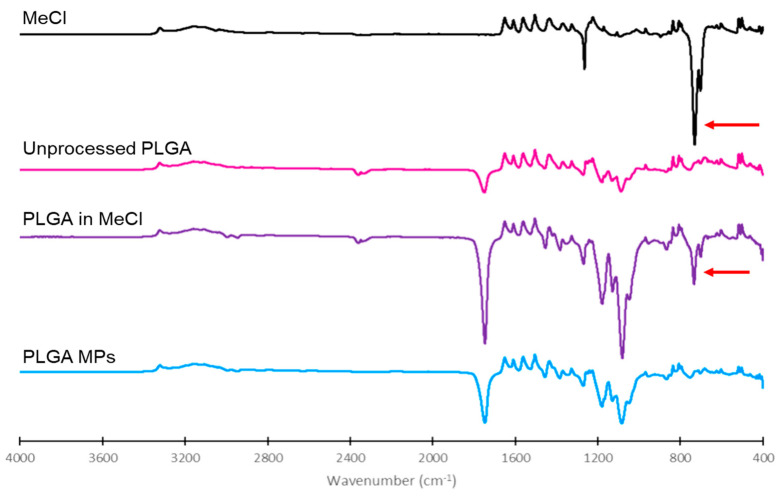
FTIR spectra of MeCl, unprocessed PLGA, 50:50 MeCl:PLGA, and PLGA MPs. The arrows indicated the C-CL bond stretching peak at 700 cm^−1^.

The complete removal of MeCl from the final PLGA MPs was confirmed through FTIR analysis (Figure 3). The MeCl spectra are characterized by a strong, sharp peak at 700 cm^−1^, corresponding to C-Cl bond stretching. This peak is also observed in the mixture of PLGA and MeCl, but not in the samples of unprocessed PLGA or the spray-dried PLGA MPs.

#### Dispersion of PLGA MPs from Bulk Powder

**Figure 4:**
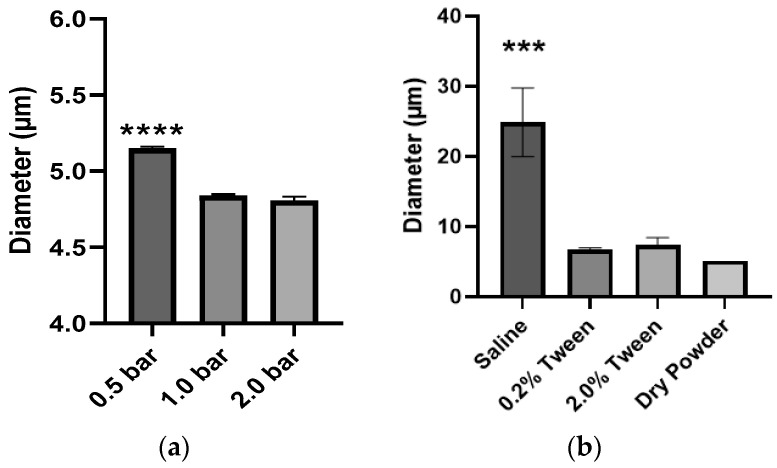
Influence of factors on the dispersion of PLGA MPs and the resulting Dv: (**a**) dispersion pressure of MPs in bulk powder, and (**b**) composition of liquid media of particles in saline suspension (mean ± standard deviation, *n* = 3, *** *p* < 0.001, **** *p* < 0.0001).

To gain insight into the extent and nature of the possible MP aggregation in the bulk powder, we evaluated the effect of three different dispersion pressure applied to the bulk powder by the RODOS disperser. The Dv decreased from 5.15 µm to 4.84 µm (*p* < 0.0001) when the dispersion pressure was increased from 0.5 bar to 1.0 bar (Figure 4a), but there was no significant decrease in Dv when the pressure was increased from 1.0 to 2.0 bar.

We further evaluated the dispersion of the MPs by suspending them in normal saline. Figure 4b shows that PLGA MPs were significantly aggregated in saline, as evidenced by the large increase in Dv (24.88 µm) compared to the Dv of MPs as a powder (5.15 µm). Therefore, Tween 80 was added to the saline solution to aid MP dispersion by decreasing the surface tension in the media. Figure 4b shows that the addition of Tween 80 led to a decrease in the Dv of MPs in suspension, from 24.88 µm to 6.71 µm with 0.2% *v*/*v* Tween 80 (*p* < 0.0001), which was not significantly different from the Dv of the bulk powder. Increasing the concentration of Tween 80 in saline did not result in an additional decrease in Dv.

### 3.2. Aerosol Performance

**Figure 5:**
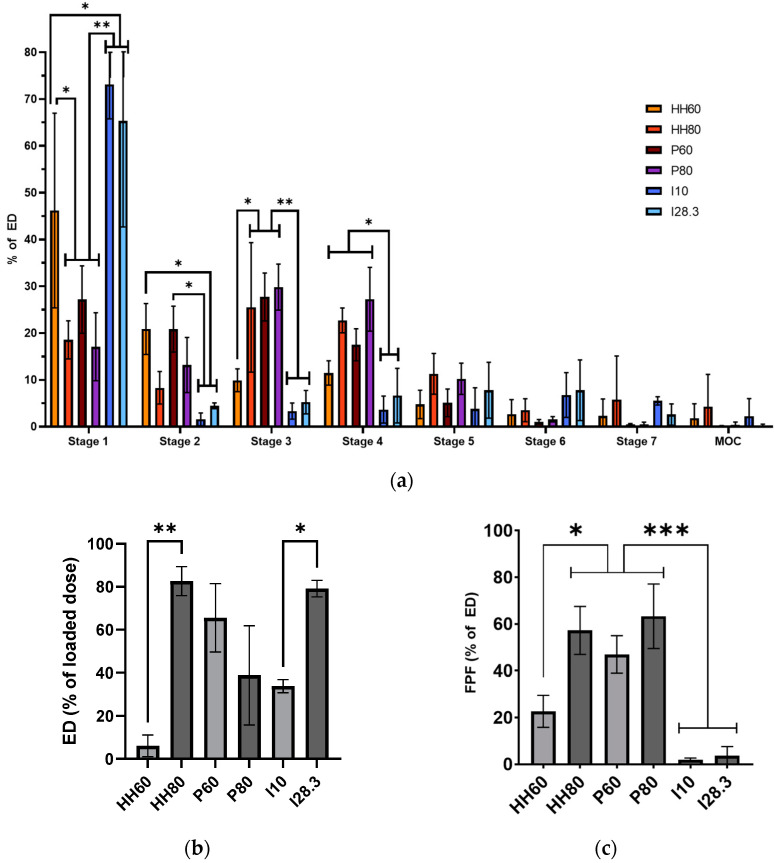
Influence of the device and flow rate on the aerosol performance of Nile red PLGA MPs from dry powder analyzed with (**a**) deposition pattern in the collection cups of the NGI after the dispersion from the HandiHaler at 60 (HH60) and 80 L/min (HH80), the Plastiape at 60 (P60) and 80 L/min (P80), or the Insufflator^®^ at 10 (I10) or 28.3 L/min (I28.3) (two-way ANOVA and Tukey’s correction for multiple comparisons); (**b**) emitted dose (ED); and (**c**) fine particle fraction (FPF) of MPs. Data presented as mean ± standard deviation (*n* = 3, * *p* < 0.05, ** *p* < 0.01, *** *p* < 0.001).

Figure 5a shows the aerosol performance of Nile red PLGA MPs following dispersion from dry powder as a function of the device and flow rate. Most of the MPs dispersed by the HandiHaler at 60 L/min were deposited in stage 1 or 2, but increasing the flow rate to 80 L/min decreased the proportion of MPs deposited in the first stages and increased the proportion of MPs in the respirable range (stages 3 and 4). MP dispersion with the Plastiape device was also dependent on flow rate, but the fraction deposited in stage 1 was significantly smaller than that after dispersion with the HandiHaler, and thus, a larger fraction of MPs were deposited in stages 3 and 4 of the NGI. In contrast, the majority of MPs dispersed with the Insufflator^®^ were deposited in stage 1, regardless of the flow rate.

The flow rate greatly affected the ED of Nile red PLGA MPs upon dispersion with the different devices (Figure 5b), with a larger ED from the HandiHaler (*p* = 0.015) and Insufflator^®^ (*p* = 0.0151) at the higher flow rate, and from the Plastiape at the lower flow rate (non-significant). The FPF (Figure 5c) was 57.21% for the HandiHaler at the high flow rate, similar to the FPF of the Plastiape at the low and high flow rates (46.94% and 63.31%, respectively). Lowering the flow rate for the HandiHaler resulted in a reduction of FPF from 57.21% to 22.66% (*p* = 0.003). Notably, the dispersion with Insufflator^®^ also had a comparatively low FPF at both flow rates (1.81% at 10 L/min and 3.62% at 28.3 L/min, *p* < 0.001).

**Figure 6:**
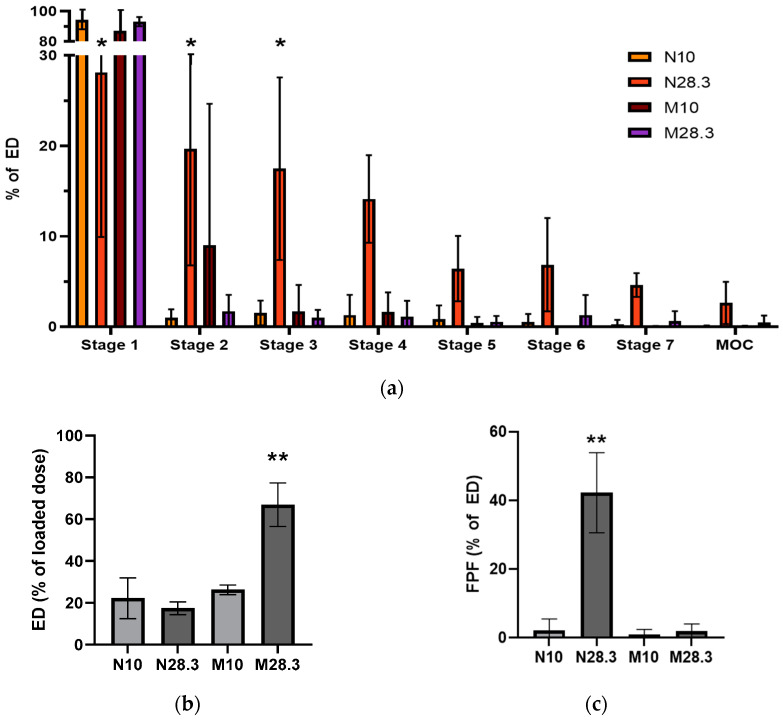
Influence of the device and flow rate on the aerosol performance of Nile red PLGA MPs in aqueous suspension. (**a**) Deposition pattern in the collection cups of the NGI after aerosol generation from the MicroSprayer^®^ at 10 (M10) or 28.3 L/min (M28.3) or the nebulizer at 10 (N10) or 28.3 L/min (N28.3) (two-way ANOVA and Tukey’s correction for multiple comparisons), (the *Y*-axis is broken between the 30% and 80% to highlight the differences between the different stages of the NGI), (**b**) emitted dose (ED), and (**c**) fine particle fraction (FPF)F of MP suspension. Data presented as mean ± standard deviation (*n* = 3, * *p* < 0.05, ** *p* < 0.01).

Figure 6 shows the aerosol performance of Nile red MPs suspended in saline with 0.2% (*v*/*v*) Tween 80 as a function of the device and the flow rate. Most droplets generated by the MicroSprayer^®^ at both flow rates and by the nebulizer at the low flow rate were deposited in stage 1, and only a smaller fraction of the droplets were deposited in the other stages (Figure 6a). In contrast, droplets generated from the MP suspension with the nebulizer at the higher flow rate were more uniformly deposited in all stages of the NGI.

The ED of the wet aerosol devices was comparable, apart from the MicroSprayer^®^ at the higher flow rate (66.97%, *p* < 0.001). The FPF of the wet aerosol devices was low (<3%), except for the nebulizer at the higher flow rate, which had an FPF of 55.71% (*p* = 0.002). Interestingly, the increased ED of the MicroSprayer^®^ at the high flow rate did not result in more than a modest increase in FPF, while the increased FPF of the nebulizer at the higher flow rate was not correlated with an increased ED.

**Figure 7:**
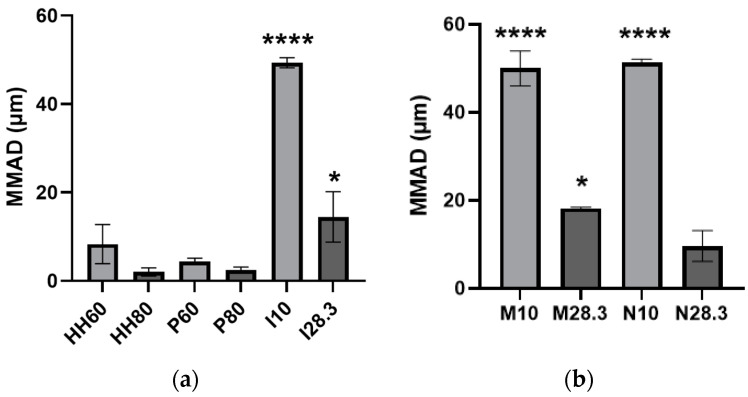
MMAD of PLGA MPs (**a**) emitted as dry powder from the HandiHaler at 60 (HH60) and 80 L/min (HH80), the Plastiape at 60 (P60) and 80 L/min (P80), or the Insufflator^®^ at 10 (I10) or 28.3 L/min (I28.3) and (**b**) as an MP suspension emitted from the MicroSprayer^®^ at 10 (M10) or 28.3 L/min (M28.3) or the nebulizer at 10 (N10) or 28.3 L/min (N28.3). Data presented as mean ± standard deviation (*n* = 3, * *p* < 0.05, **** *p* < 0.0001).

Based on the NGI data for the wet and dry aerosols, aerodynamic diameter (MMAD) was calculated (Figure 7). The particles with the smallest MMAD were emitted from the HandiHaler and Plastiape devices at the higher flow rates (2.05 ± 0.75 µm and 2.50 ± 0.53 µm, respectively). The particles with the highest MMAD were emitted from the Insufflator^®^, MicroSprayer^®^, and nebulizer at the lower flow rate (49.3 ± 0.94, 50.0 ± 3.24, and 51.3 ± 0.62 µm, respectively).

### 3.3. PLGA MP Deposition In Vivo

**Figure 8:**
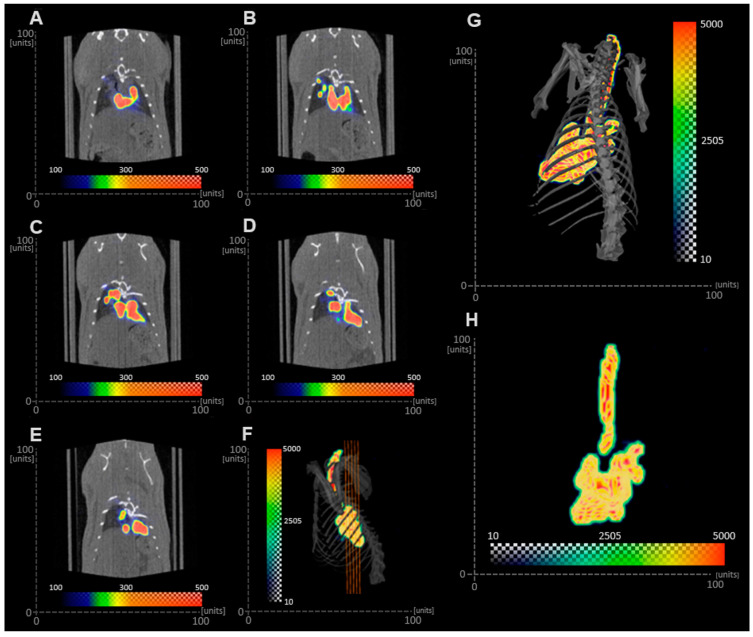
Deposition of radiolabeled MPs suspension in rat lungs. (**A**–**E**) Orthogonal slices of rat chest with CT image (black and white) overlaid with SPECT activity (color). (**F**) Locations of slices. (**G**) Dorsal view of voltex of 99Tc-particle activity (color) with isosurface of rat skeletal structure from CT (grey). (**H**) Voltex of 99Tc-particle activity (color).

The deposition of the Tc-99m-labeled PLGA MP suspension in the airways of rats can be seen in Figure 8. Orthogonal slices of the lungs (Figure 8A–G) confirm that the MPs deposited in the different regions of the respiratory tract. The SPECT activity (Figure 8H) shows that particle deposition occurred throughout the entire airways, including the trachea and larger airways. The ED of the Tc-99m-labeled PLGA MP suspension from the MicroSprayer^®^ was approximately 60%. A low percentage of activity was found in the trachea and bronchi. The deposition in the trachea and bronchi was equivalent to 0.2% of the ED, and in the bronchioles was 1.5% of the ED. There was no significant deposition in the alveoli.

## 4. Discussion

Despite the many advantages of the pulmonary route to treat respiratory diseases, it is still often considered inefficient or impractical for clinical applications other than CF, COPD, and asthma. Preclinical evaluation of novel inhaled therapies is complex because there are many factors that need to be considered to determine what are the key in vitro determinations that should be performed. The manner in which the results of those determinations are interpreted or applied to design in vivo studies or run in silico simulations is challenging, but their integration in terms of therapies for humans is even more challenging. The present study aimed to provide a basic overview of items to be considered in the design, performance, and interpretation of in vitro and in vivo studies to assess the suitability of formulating novel compounds for inhaled therapies and to inform in silico models that will help to design better-inhaled therapies.

To optimize their site of deposition, we first optimized the parameters to manufacture PLGA MPs in respirable sizes using DoE1. The outcomes measured were Dg and Dv and their corresponding GSDs. The Dg of particles prepared in DoE1 ranged from 2.7 to 3.7 µm, whereas their corresponding Dv was 1.5 to 2-fold larger, indicating that the MPs were aggregated in the bulk powder. Therefore, DoE2 was designed with the aim of preparing MPs with no aggregation. The aspiration rate was selected as a variable because the DoE1 cube plot and Equation (2) (Figure 1) indicated that an increase in aspiration rate would decrease Dg and Dv. However, the aspiration rate could only be increased to 35 m^3^/min due to equipment limitations. The feed flow rate was also selected as a variable for DoE2 because it was hypothesized that reducing the feed flow rate would reduce the size of the droplet produced by the atomizer, which would reduce the size of the spray-dried particles. Still, the Dv of the resulting MPs was only reduced modestly (5.07–5.26 µm), most likely due to a comparable reduction in Dg (2.30–2.70 µm).

To determine if PLGA MPs could be dispersed with sufficient external pressure provided by either the aerosolizing device or the inspiratory pressure of the patient, we evaluated three dispersion pressures applied to PLGA MPs as dry powders in bulk by the HELOS/RODOS apparatus. The Dv of MPs decreased from 5.15 to 4.84 µm when the dispersion pressure was increased from 0.5 to 1.0 bar (Figure 4a), but an increase of pressure to 2.0 bar did not result in a significant reduction of Dv of the MPs. This suggested that while some aggregation may be due to electrostatic forces, there were other forces governing the interactions between MPs. The PLGA MPs were also dispersed in saline solution (Figure 4b), but the Dv of MPs in suspension was even larger (24.88 µm) than in dry powder (5.15 µm), perhaps due to the surface tension of the liquid media (saline). This hypothesis was confirmed because the addition of a surfactant (0.2% Tween 80) to the suspension resulted in a reduction of Dv from 24.88 µm to 6.71 µm. However, the Dv of MPs in suspension was not reduced further upon the addition of an extra Tween 80 to the suspension media. This data, in conjunction, illustrates the importance of selecting the appropriate manner and media to disperse therapeutic MPs and verifying the influence of these two factors on the resulting Dv before administering them to animals or patients.

The third and most important in vitro evaluation is the aerosol performance of PLGA MPs upon aerosolization using different devices for use in animals and patients. Because the same PLGA MP formulation was used with each device, the differences in aerosol performance reported here are attributed to the device instead of particle characteristics. The Insufflator^®^ was evaluated to determine the deposition pattern of MPs after direct administration to an animal, whereas the HandiHaler and Plastiape devices were evaluated to determine the deposition of the MPs in patients. As expected, the dry MPs showed a better aerosol performance (higher %ED and FPF) at a high flow rate (80 L/min) upon dispersion with the HandiHaler (Figure 5), a high-resistance device [28]. Conversely, the dispersion of MPs at a low flow rate (60 L/min) with the Plastiape device, a low resistance device, resulted in a larger %ED, but the FPF was slightly smaller at a higher flow rate (80 L/min). These results highlight the importance of evaluating DPIs with low and high resistance at low and high flow rates to make a better-informed decision on what device should be paired with a novel inhaled formulation for the best therapeutic effect in a certain patient population.

For animal studies, it is important to properly interpret the data of aerosol performance generated with devices for direct administration (Insufflator^®^ and Micro -Sprayer^®^). For example, the %ED of MPS dispersed with the Insufflator^®^ at a higher flow rate was similar to that of the two DPIs evaluated (Figure 5b). However, most of the MPs were deposited on the first stage of the NGI (73.14% at 10 L/min and 65.31% at 28.3 L/min), reflected in the very small FPF obtained at the two flow rates tested. This data could be mistakenly interpreted as that all MPs would be deposited in the trachea of a rat. Nevertheless, it is important to understand that administration of powders with the Insufflator^®^ requires the insertion of the tube of the Insufflator^®^ into the trachea of the animal and the powder aerosolized with an external source of air. Thus, this procedure bypasses the oropharyngeal and tracheal deposition of powders, essentially “forcing” the MPs into the smaller airways and alveoli of the animal, as shown in several publications [29,30,31]. Nevertheless, it is of paramount importance to note that the deposition of MPs after direct administration with either the Insufflator^®^ or Micro -Sprayer^®^ is highly dependent on the skill and experience of the person performing the administration as well as the speed and pressure used with the syringe used to disperse the MPs into the animal’s airways [32].

We also evaluated the aerosol performance of PLGA MPs in suspension using the MicroSprayer^®^ or the Hudson nebulizer because researchers often use MPs suspended in saline or another liquid media to generate the aerosols used in preclinical studies when they do not have access to a dry powder disperser or have a limited amount of material. This was the case in the present study, where the method used to radiolabel the PLGA MPs for in vivo studies used liquid media and produced radiolabeled MPs in suspension. Aerosols generated from PLGA MPs in suspension with the MicroSprayer^®^ showed a large fraction of the aerosol deposited on stage 1 of the NGI (87.16% at 10 L/min and 93.22% at 28.3 L/min, Figure 6a). Surprisingly, the %ED from the MicroSprayer^®^ at 28.3 L/min, was higher than that of the nebulizer at any flow rate (Figure 6b), but the FPF of aerosols from the MicroSprayer^®^ were significantly lower than those from the nebulizer (Figure 6c), which correlates with significant aerosol deposition on stages 3–7 of the NGI (Figure 6a). The reason for the low %ED from the nebulizer was investigated by measuring the Dv of the droplets emitted from the nebulizer using different liquids, including saline with or without different concentrations of Tween 80. Determination of the Dv by laser diffraction revealed that the size of the droplets was 5.32 ± 0.21 µm, regardless of the solvent or suspension composition. By comparing the droplet size of liquid alone to the size of the PLGA MPs in suspension (Figure 4b), we determined that the droplets were too small to include all the MPs in the suspension into the aerosol generated, even in the presence of Tween 80. Thus, it is likely that only the smallest MPs in the suspension could be carried by the droplets (<35%), which would explain the low %ED observed for the nebulizer (13.13–32.46%, Figure 6b). These results highlight the importance of performing a suitable in vitro characterization of aerosol deposition and interpreting these results in the context of the procedure performed in vivo.

Figure 7 shows the MMAD of the aerosols emitted from all evaluated devices. As expected from their deposition patterns on the stages of the NGI, aerosols emitted from direct administration devices and nebulizers at low flow rates had the largest MMAD, but they decreased when these devices were evaluated at a higher flow rate (Figure 7). Only the HandiHaler and the nebulizer evaluated at high flow rates, as well as the Plastiape evaluated at both flow rates, emitted aerosols with MMADs in the respirable sizes for humans. From these devices, only the nebulizer could be used to administer aerosols to awake, spontaneously breathing animals unless direct administration methods are employed, which would require sedating/anesthetizing the animal. Based on the results of the present study with nebulized MPs, only a limited amount of the aerosol would be deposited in the different regions of their respiratory tract.

The importance of selecting the appropriate expression of diameter (Dg, Dv, or MMAD) from in vitro studies to predict the sites of aerosol deposition in the respiratory tract of humans or rats in the absence of inspiratory flow rate is illustrated in Table 3 and Table 4, respectively. Based on the diameters of human or rat airways obtained from the literature [33,34,35,36,37,38,39,40,41], we predicted the approximate fractions of the dose that would be deposited in the different regions of the respiratory tract. For instance, if Dg was employed to predict the deposition of MPs in human studies without considering the Dv of particles or the device employed to aerosolize the MPs, it could be incorrectly hypothesized that 23.93% and 68.51% of MPs would be deposited in the alveolar and bronchiolar regions of a patient, respectively (Table 3, first row). Alternatively, if only Dv were considered, it could be wrongly assumed that dry MPs would deposit almost equally in the bronchi and bronchioles (Table 3, second row). Instead, when the aerosol performance of MPs in the device is considered at different flow rates of the NGI, it can be predicted more accurately that a larger fraction of the MPs dose would be deposited in the alveoli and bronchioles with the HandiHaler or Plastiape devices. Moreover, a comparison of aerosol performance between devices would reveal that the Plastiape device may deliver a more uniform dose across patients with different inspiratory forces. This illustrates the importance of considering the flow rate at which the NGI is operated and the resistance of the device when predicting the site of aerosol deposition.

On the other hand, if deposition studies were to be performed in rats with MPs administered by passive inhalation and the sites of aerosol deposition were to be predicted using only Dg, it could be hypothesized that 4.79% and 19.14% of the MPs dose would be deposited in the alveolar and bronchiolar region of the rat, respectively (Table 4, first row).

Under the conditions described in the present work, the only option to deliver MPs to rats by passive inhalation would be by nebulization, and hence, since rats are obligated-nose breathers, it is of paramount importance to note the cutoff diameter of particles inhaled by the rat’s nose. Thus, a more accurate prediction would be that the fractions of the dose deposited in the alveoli, bronchioles, and bronchi would be 2.64, 11.47, and 6.44%, respectively (Table 4, last row).

If MPs were to be given by direct administration, either with the Insufflator^®^ or MicroSprayer^®^, the cross-sectional area of the airways (bronchi and bronchioles) should be considered because the aerosol is “forced” into the airways using an external source of air. Under this assumption, it would be expected that MPs administered in this manner would all be deposited in the alveolar region if only Dg or Dv were considered (Table 4, top right rows). On the contrary, the SPECT/CT images obtained from the in vivo study indicated that the Tc-99m-labeled PLGA MP suspension deposited in the bronchi and bronchioles. The discrepancy between the in vitro determination and the in vivo study can be due to different factors, including the proficiency of the person administering the MPs with the MicroSprayer^®^ or anatomical and physiological variables in the animal. The lack of training of a person using the MicroSprayer^®^ could lead to the generation of larger droplets due to a slow actuation or to inertial impaction of the droplets in the bronchi and bronchioles due to the high velocity of the droplets generated. Furthermore, since the rat was intubated and the radiolabeled MP in suspension was administered with the MicroSprayer^®^, deposition of MPs in the trachea was not expected, as animals were held in the upright position for 1 min after aerosol administration. However, the imaging procedure was performed with the animals in the supine position, so it is likely that some radiolabeled MPs were present in the trachea as a result of mucociliary clearance during the 30 min imaging procedure [19]. It is important to note that particle clearance is highly dependent on the animal model [42]. Brain and Mensah studied the rate of gold particle clearance in different species and reported that hamsters cleared particles faster than rats, followed by rabbits and mice [43]. These results highlight the importance of selecting the appropriate expression of diameter (Dg, Dv, or MMAD) from in vitro studies, the dimensions of the airways of the subject employed in the study, and physiological variables to inform the prediction of the sites of aerosol deposition in in vivo studies.

Perhaps the main disadvantages of performing a comprehensive in vitro and in vivo characterization of inhalable therapies are the cost and time, but the use of in silico studies may help to streamline some of the variables. For example, Zhao J. et al. conducted an in silico study to determine the effect of carrier shape (spherical or elongated) on the regional deposition of a drug-carrier blend emitted from the HandiHaler and a generic DPI using variable flow rates (30–90 L/min) [44]. Using sperical particles, their simulation indicated that actuation of the HandiHaler at 60 L/min resulted in an ED of about 90%, with regional deposition of drug particles of approximately 55%, 10%, and 35% in generations (G) 0–2, 3–13, and 14–23, respectively. Even though the same device and flow rate were used in the present study, the ED obtained with the PLGA MPs was much smaller (6.09%). This difference in ED could be due to several factors, including the formulation, the type of diameter considered, and the amount/volume of powder contained in the capsule that was loaded in the HandiHaler. Drug-carrier blend formulations include additional variables such as the homogeneity of the blend and the stripping of the drug particles from the carrier compared to engineered particles. The drug-carrier blend had median equivalent volume diameters of 46.0 µm and 2.8 µm for the lactose carrier and API, respectively. The GSDs of the particles were not provided, but the Dv was similar to the Dg of PLGA MPs and 1.8-times smaller than the Dv of the particles described in this study, one of them being the amount/volume of powder contained in the capsule that was loaded in the HandiHaler. While the model by Zhao et al. considered that the powder in the capsule would be approximately 10%, the body of the capsule in our study was filled, leaving only the top part void. Thus, it may be possible that in our study, the reduced void space left in the capsule was insufficient to fluidize the powder to be aerosolized and emitted from the device. In addition, while our study employed an engineered particle containing a dye and excipient in the same particle, the study by Zhao et al. considered a blend in which the drug was much smaller than the carrier, and thus the ED is higher because it is based only on the drug proportion. These differences may have also affected the regional deposition obtained in both studies. While in the study by Zhao et al., the deposition of particles in the upper airways (G0-G2) was lower (55%) than in our study with PLGA MPs (G0 = 67.07%, Table 3, 4th row), the deposition in the lower airways and alveolar region (G14-G23) was higher (35%) than in our study with PLGA MPs (G17-G23 = 7.15%). Another difference between the present study and that of Zhao et al. was that they concluded that lower flow rates were favorable to increasing the overall delivery efficiency, quantified as the number of particles depositing below G13 compared to the number of particles entering the mouth. In contrast, the present study indicates that alveolar deposition (represented by FPF) increases with increasing flow rate, which is consistent with other publications stating that the HandiHaler requires higher resistance than other DPIs to generate respirable aerosols [28].

Hayeti et al. modeled the deposition of monodispersed aerosol droplets ranging from 1 to 15 µm in rat and human airways after passive nasal inhalation using in silico simulations to develop novel scale-up correlations [45,46]. Their simulation performed in the rat model indicated that when the monodispersed aerosol droplets were 2 µm, 15% of the dose would deposit in the extra-thoracic region (ET), and 85% of the dose would deposit in the tracheobronchial (TB) tree, whereas for 4 µmm droplets, the fractions deposited in the ET and TB regions would be 65% and 35%, respectively. Even though Hayeti et al. did not describe the method by which aerosol droplets were generated, it could be assumed that it was a nebulizer, and thus, we could compare their results with the ones in the present study obtained at 28.3 L/min. These hypothesized percentages (Table 4, last row) were calculated keeping in mind that rats are obligated nose breathers and that the cutoff diameter in the nose of rats is approximately 3 µm [32]; thus, only droplets of 2 µm are compared. Under these assumptions, we hypothesized that the fractions deposited in the ET and TB regions would be 14.11% and 85.89%, respectively, which are very different from those obtained in silico by Hayeti et al. These drastic differences can be due to several factors, including the composition of the droplet (water versus a PLGA suspension), the type of diameter considered (undefined versus MMAD), the droplet size distribution (monodisperse versus heterodisperse) as well as the regions considered (ET and TB versus alveolar, bronchiolar, bronchial and tracheal). However, the main difference was that in our predicted deposition, we considered the cutoff diameter in the nose of rats. These differences in predicting aerosol deposition in a laboratory animal highlight the importance of incorporating key anatomical and physiological features such as the animal being an obligated nose breather and the cutoff diameter of its nose.

## 5. Conclusions

**Figure 9:**
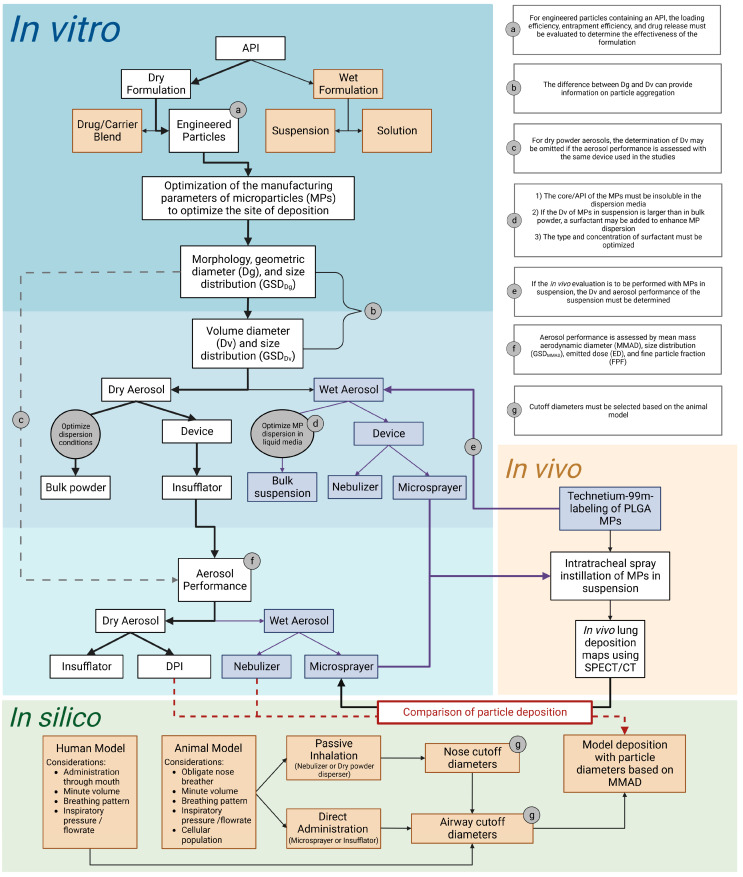
Summary of the elements that should be considered when designing and performing the in vitro and in vivo characterization of an inhalable formulation, as well as the proper parameters that should be used to inform in silico modeling. In the blue region, the items in white squares that follow the direction of the bold black arrows summarize the determinations illustrated with examples in the present study. These bold black arrows represent the order that we recommend for the performance of each determination. The items in the purple squares and bold purple arrows represent the additional in vitro determinations that had to be performed to accommodate the liquid formulation of radiolabeled MPs provided to perform the in vivo deposition study (orange region). Lastly, in the green region, we provided a summary of the anatomical and physiological variables that should be considered for the design of in silico studies.

The present study provided a basic overview of the elements that should be considered when designing and performing the in vitro and in vivo characterization of an inhalable formulation, which is summarized, together with our recommendations, as a flow diagram in Figure 9. The manner in which the results of the in vitro determinations should be applied to the design and interpretation of in vivo studies was illustrated with examples. The use of the appropriate elements of the in vitro characterization (Dg or Dv versus MMAD, passive inhalation versus direct administration) is also illustrated. The proper integration of the in vitro, in vivo, and in silico studies should provide more accurate information about the potential of a novel inhaled therapy to treat pulmonary diseases.

## Figures and Tables

**Table 1:** Spray-drying parameters in DoE1 to optimize the sizes of PLGA MPs, the resulting diameters (Dg, Dv), and the corresponding GSDs.

	Manufacturing Variables			Particle Size			
Standard Run	Feed Concentration % (*w*/*v*)	N_2_ Flow Rate L/h	Aspiration Rate m^3^/min	Geometric Diameter		Volume Diameter	
				Dg	GSD	Dv	GSD
1	0.25	742	30	2.70	1.54	5.40	1.16
2	0.35	742	30	2.85	1.48	5.47	1.17
3	0.25	600	20	3.50	1.67	5.68	1.20
4	0.35	742	20	3.00	1.68	5.43	1.16
5	0.35	600	20	3.10	1.75	5.57	1.18
6	0.25	742	20	3.30	1.94	5.63	1.18
7	0.35	600	30	3.70	1.60	5.39	1.15
8	0.25	600	30	3.15	1.62	5.51	1.18

**Table 2:** Spray-drying parameters entered in DoE2 to refine the size and distribution of PLGA MPs, the resulting diameters (Dg, Dv), and the corresponding GSDs.

	Manufacturing Variables		Particle Size			
Standard Run	Aspiration Rate m^3^/min	Feed Rate mL/min	Geometric Diameter		Volume Diameter	
			Dg	GSD	Dv	GSD
1	30	3.0	2.55	1.64	5.25	1.20
2	35	3.0	2.35	1.56	5.07	1.15
3	35	7.5	2.30	1.68	5.10	1.15
4	30	7.5	2.70	1.78	5.26	1.16

**Table 3:** Percentage of PLGA MP that would be predicted to deposit in the different regions of the human airways when based on a particular measured diameter (Dg, Dv, or MMAD).

Measurement	Alveoli ^1^ <2 µm	Bronchioles ^1^ 2–5 µm	Bronchi ^1^ 5–7 µm	Trachea ^1^ 7–10 µm
Geometric diameter (Dg)	23.93	68.51	6.30	0.00
Volume diameter (Dv, bulk powder)	0.00	46.46	50.85	2.69
Volume diameter (Dv, powder in suspension)	3.51	27.14	11.79	57.56
Mean Mass Aerodynamic Diameter (MMAD)				
HandiHaler (60 L/min)	7.15	15.51	10.27	67.07
HandiHaler (80 L/min)	16.75	40.46	24.23	18.56
Plastiape (60 L/min)	6.09	40.85	5.04	48.02
Plastiape (80 L/min)	11.57	51.47	19.87	17.09
Nebulizer (10 L/min)	0.03	0.15	0.67	99.15
Nebulizer (28.3 L/min)	12.19	5.53	16.96	65.32

^1^ Alevoli corresponds to generations 17–23 of the Weibel lung model; Bronchioles correspond to generations 5–17; Bronchi correspond to generations 1–4; Trachea corresponds with generation 0.

**Table 4:** Percentage of PLGA MP that would be predicted to deposit in the different regions of the rat airways after passive inhalation when based on a particular measured diameter (Dg, Dv, or MMAD).

Measurement	Passive Inhalation			
	Alveoli ^1^ <1 µm	Bronchioles ^1^ 1.0–2.0 µm	Bronchi ^1^ 2.01–3 µm	Trachea ^1^ >3 µm
Geometric diameter (Dg)	4.79	19.14	40.30	35.77
Volume diameter (Dv, bulk powder)	0.00	0.00	0.00	100.00
Volume diameter (Dv, MPs in suspension)	1.28	2.23	6.05	90.44
Mean Mass Aerodynamic Diameter (MMAD)				
Nebulizer (10 L/min)	0.00	0.05	0.28	99.67
Nebulizer (28.3 L/min)	2.64	11.47	6.44	79.45

^1^ Alevoli corresponds to generations 17–23 of the Weibel lung model; Bronchioles correspond to generations 5–17; Bronchi correspond to generations 1–4; Trachea corresponds with generation 0.

## Data Availability

All the relevant data are available from the corresponding author upon request.
